# The Effect of Granular Commercial Fertilizers Containing Elemental Sulfur on Wheat Yield under Mediterranean Conditions

**DOI:** 10.3390/plants8010002

**Published:** 2018-12-20

**Authors:** Dimitris L. Bouranis, Dionisios Gasparatos, Bernd Zechmann, Lampros D. Bouranis, Styliani N. Chorianopoulou

**Affiliations:** 1 Plant Physiology and Morphology Laboratory, Crop Science Department, Agricultural University of Athens, 11855 Athens, Greece; lampros.bouranis@ucdconnect.ie (L.D.B.); s.chorianopoulou@aua.gr (S.N.C.); 2 Soil Science Laboratory, Department of Hydraulics, Soil Science and Agricultural Engineering, School of Agriculture, Faculty of Agriculture, Forestry and Natural Environment, Aristotle University of Thessaloniki, 54124 Thessaloniki, Greece; gasparatos@agro.auth.gr; 3 Center for Microscopy and Imaging, Baylor University, One Bear Place 97046, Waco, TX 76798-7046, USA; Bernd_Zechmann@baylor.edu

**Keywords:** elemental sulfur, granular S containing fertilizers, durum wheat, yield, NBPT

## Abstract

The demand to develop fertilizers with higher sulfur use efficiency has intensified over the last decade, since sulfur deficiency in crops has become more widespread. The aim of this study was to investigate whether fertilizers enriched with 2% elemental sulfur (ES) via a binding material of organic nature improve yield when compared to the corresponding conventional ones. Under the scanning electron microscope, the granules of the ES-containing fertilizer were found to be covered by a layer of crystal-like particles, the width of which was found to be up to 60 μm. Such a layer could not be found on the corresponding conventional fertilizer granules. Several fertilization schemes with or without incorporated ES were tested in various durum wheat varieties, cultivated in commercial fields. The P-Olsen content of each commercial field was found to be correlated with the corresponding relative change in the yields (YF/YFBES) with a strong positive relationship. The content of 8 ppm of available soil phosphorus was a turning point. At higher values the incorporation of ES in the fertilization scheme resulted in higher yield, while at lower values it resulted in lower yield, compared with the conventional one. The experimental field trials that established following a randomized block design, were separated in two groups: One with P-Olsen ranging between 18–22 ppm and the other between 12–15 ppm, the results of which corroborated the aforementioned finding. The use of ES in all portions of fertilization schemes provided higher relative yields. The coexistence of ES with sulfate in the granule was more efficient in terms of yield, when compared to the granule enriched with ES alone under the same fertilization scheme and agronomic practice. The application of fertilizer mixtures containing the urease inhibitor N-(n-butyl) thiophosphoric triamide (NBPT), ES and ammonium sulfate resulted in even higher relative yields. Yield followed a positive linear relationship with the number of heads per square meter. In this correlation, the P-Olsen content separated the results of the two groups of blocks, where the applied linear trend line in each group presented the same slope.

## 1. Introduction

Sulfur (S) deficiency in crops has become a common agronomic issue in many countries, mainly due to reduced atmospheric inputs and the use of sulfur free high-analysis fertilizers (i.e., fertilizer products that contain a high percentage of nutrients, usually more than 30 per cent). The development of S deficiency in plants during the last decades has been discussed in detail [1,2]. Therefore, the demand for and interest in S fertilizers has been intensified. Wheat (*Triticum aestivum*) is the most widely cultivated crop in the world, a crop that has a relatively low requirement for S. However, S deficiency in wheat has become increasingly widespread [3].

A detailed discussion of S fertilizers has been provided by Grant and Hawkesford [4]. The most important inorganic S fertilizers are ammonium sulfate (AS, 24% S), gypsum (18% S), single superphosphate (12% S) and elemental sulfur (ES, 100% S) [5]. The granular fertilizers that provide S when applied in crops are mainly based on sulfate salts, because sulfate-S (i.e., S in the form of sulfate) is readily available to plants, however highly mobile in the soil profile.

On the other hand, ES, a soil amendment with a long history in agricultural practice, is insoluble in water and works as a slow release S fertilizer, which becomes available to plants after oxidation to sulfate by microorganisms [6]. According to Boswell and Friesen [7], 80% of ES (particles of 75–150 μm) was found to be oxidized after six months of application in a warm temperate region of New Zealand. The incorporation of ES into the granules of commercial fertilizers provide an efficient use of ES in agriculture, as S component in the fertilization management practices [8].

In a recent industrial approach, the granules of conventional fertilizers (F) were enriched with 2% ES (*w*/*w*) via a binding material of organic nature (B, i.e., a mixture of molasses and glycerol) (FBES technology, [9]). The aforementioned approach for the production of granular fertilizers of this type can be applied to various granular fertilizers, excluding nitrates, thus providing a family of ES-enriched fertilizers.

While several studies have been conducted to assess the efficiency of ES as fertilizer, there are scarce results from agronomic field trials on the granular ES-enriched macronutrient fertilizers, in order to estimate if these products may have a positive effect on crop production [10]. ES-enriched urea (U) produced by various technological approaches is used as fertilizer over the last 25 years. On the other hand, NBPT (i.e., N-(n-butyl)-thiophosphoric triamide) is a commonly used urease inhibitor for retarding U hydrolysis in the past 10 years [11]. U or fertilizer materials derived primarily from U, enriched with other additional useful additives and plant nutrient materials, can be treated with a solution of NBPT dissolved in one or more amino alcohols to reduce nitrogen volatilization. The solution may be applied as a coating for granular U fertilizers [12].

The initial field experiment showed that the commercial ES-fertilizers enriched with the FBES technology increased yield of durum wheat (*Triticum durum* or *Triticum turgidum* subsp. *durum*) crop by 27.3% compared with the corresponding conventional ones [13]. In order to verify whether the fertilizer products of the aforementioned FBES technology were able to provide a higher yield under the common fertilization schemes and agricultural practices, commercial field trials with durum wheat crops were established and monitored in central and northern Greece. In these schemes, U was the common N fertilizer. Furthermore, U in mixtures with AS (UAS) was also of common use. Both U and UAS can be enriched with ES by the FBES technology (U/ES, UAS/ES). UAS/ES can release readily available sulfate-S for immediate use by the crop, while ES can provide available S after S oxidation to sulfate-S. In order to further explain the findings obtained from the commercial field trials, experimental field trials were established and monitored. U or UAS along with their stabilized form with NBPT are also of common use in the commercial fertilization schemes (U/NBPT). No product was available containing both of the aforementioned chemicals, i.e., ES and NBPT. Was the use of U/NBPT or UAS/NBPT compatible with the use of U/ES or UAS/ES? In order to verify to what degree the enriched fertilizer schemes with ES and NBPT were influencing the yield, the following question was addressed: Could a mixture of both fertilizer types be an effective one?

## 2. Materials and Methods

### 2.1. Commercial Field Trials

In the wheat growing season of 2015–2016, several crops with durum wheat were established in different areas of central and northern Greece (Groups A1 and A2). Sowing and harvesting took place mid November 2015 and end June 2016 respectively. In 2016–2017, more crops were established in the same or other fields (Group A3). Sowing and harvesting took place mid November 2016 and end June 2017 respectively. All fields were of commercial use. Each production field was divided into two equal areas; one of them was subject to conventional fertilization treatment according to the local agricultural practices (F-trial), while the other one received the corresponding FBES-trial. The fertilization scheme and the agronomic details per trial (i.e., area in ha, sowing rate, cultivar, fertilizer type and rate) are provided in Table 1, along with the percentage relative yield. Soil fertility of each field was determined prior to crop establishment. In order to ensure comparable soil conditions between the corresponding F- and FBES-trials, each area was arbitrarily divided into plots 15 m × 7 m (105 square meters) each [14]. All perimetric plots were excluded, while the internal plots were grouped into five successive groups. Within each group of plots, one composite sample per group (0.5 kg) was collected at the depth of 0–20 cm, these samples were pooled together and a final composite sample per trial (2.5 kg) was formed and analyzed. Soil analysis data and yields are provided in Table 2. Only the fields with comparable F- and FBES-trials with regard to soil fertility are further analyzed and discussed in this study.

### 2.2. Experimental Field Trials

Based on the experience gained during the seasons 2014–2015 and 2015–2016, two experiments with durum wheat crops (cv. Simeto) were established in the experimental fields of the Agricultural University of Athens in Aliartos at Viotia county during the season 2016–2017, based on a randomized block design. Each plot was 18 square meters and 1 m apart from the next ones. In each plot of the provided area for experimentation, the P-Olsen content was determined and two areas of almost 0.2 ha each were located, one with P-Olsen values ranging between 18–22 ppm (Group B1) and the other between 12–15 ppm (Group B2). Soil samples were collected at depth of 0–20 cm prior to the crop establishment and fertilizer application. The plot that received ammonium phosphate (16-20-0-13S) as initial fertilizer (starter) and urea (U; 46-0-0) as additional fertilizer (topdressing) served as the reference crop. The compatibility of urea-FBES (U + 2% ES; U/ES) or urea ammonium sulfate (UAS + 2% ES; UAS/ES) along with the corresponding ones containing the urease inhibitor NBPT (U/NBPT) or urea ammonium sulfate (UAS/NBPT) was also studied in this experiment. Each combination was repeated three times.

### 2.3. The Nature of the FBES Granules

The FBES granules tested in this research (16-20-0/ES, 20-10-0/ES, 10-20-0/ES, U/ES, UAS/ES) have been prepared by Sulphur Hellas S.A. (Sulfogrow®) as it follows: The fertilizer granules were mixed initially with ES in the form of dust at a percentage of 2% to 4% (*w*/*w*, ES to fertilizer granules). The mixture passed through a shower of fine droplets consisting of a 1:1 mixture of molasses and glycerol, which acted as the binding system, in such a way that the whole surface was exposed. The binder was added at a percentage of 0.4% to 1.2% (*w*/*w*, binder to fertilizer granules). Then, the mixture was led to a mixer, where the sticky ES dust was evenly attached onto the sticky fertilizer granules, thus forming the final FBES product [9].

### 2.4. Soil Analysis

After the soil samples were collected, were air-dried and ground to 2 mm prior to analysis. The particle size analyses were conducted using the hydrometer method, with a 2-h reading for the clay content [15]. The CaCO_3_ equivalent percentage was estimated using a digital calcimeter [16]. Soil pH was measured in a 1:1 soil: Distilled water (w-v) suspension [17] and electrical conductivity (ECe) in saturation extract. Soil organic matter (SOM) was determined using the Walkley-Black wet digestion method [18] and the available P (P-Olsen) according to the Olsen method [19]. Exchangeable K (K_exch_) was determined using the ammonium acetate extraction method [20]. Extractable Fe, Mn, Cu and Zn were determined with diethylene-triamine-pentaaceticacid (DTPA) [21]. Many studies have shown that DTPA is the most widely used extractant for the determination of metal availability in soils [22,23]. The soil humic substances content (HS) was determined according to Jones (1999) [24]. The main soil properties of each field trial are presented in Table 2.

### 2.5. Head Measurements

In the experimental field trials harvesting took place on 1–3 July 2017. Per plot, all stems were collected and were placed into bags. In the laboratory, each stem was separated from its head, and the heads were weighed. Three sets of a hundred heads each were weighed, and the seeds per set were separated, collected and weighed.

### 2.6. Scanning Electron Microscopy

Cross sections of granules were made using razor blades and mounted on SEM pedestal using conductive silver epoxy paint (EMS, Hatfield, USA). Samples were then sputter coated with gold (Denton Vacuum Desk II Sputter coater, Denton Vacuum, Moorestown, USA) and observed with a JSM-5410 scanning electron microscope (JEOL, Tokyo, Japan) at a working distance of 40 mm at 10 kV.

### 2.7. Statistical Analysis

Yield levels were handled as continuous variables. A linear mixed effects (LME) model [25] was used to assess the statistical significance of the covariates on the response variable, taking into account the within-group variability that exists for the repeated measurements setting. Analysis of variance (ANOVA) was performed on the LME model to determine which of the model covariates (i.e., Phosphorus level, Initial Fertilization and Additional Fertilization) had a significant effect on the average value of yield. All higher-order interactions were tried. Tests were declared significant when *p* < 0.05. All analyses were performed using the R statistical software (R Development Core Team, 2018) [26].

## 3. Results

### 3.1. Characteristics of ES Enriched Fertilizer Granules

The first product of this family of fertilizers was of the 20-10-10 type, where nitrogen was provided as ammonium sulfate, phosphorus as triple superphosphate, while potassium was provided as potassium sulfate. The diameter of ES-containing fertilizer granules (20-10-10+2% ES) was 2.463 ± 0.579 mm (mean value ± standard deviation; n = 100), while the corresponding one of the conventional fertilizer granules was 2.447 ± 0.617 mm (n = 100). The two-tailed *P*-value was 0.8502 thus by conventional criteria (t = 0.1891, df = 198, standard error of difference = 0.085) there was no statistically significant difference between the means. The granules were then categorized according to their size. The ES-fertilizer granules presented the following size distribution: <2 mm = 20%, 2–2.49 mm = 33%, 2.5–2.99 mm = 19%, 3–3.49 mm = 25%, >3 mm = 3%. The corresponding distribution of the conventional fertilizer was: <2 mm = 18%, 2–2.49 mm = 42%, 2.5–2.99 mm = 11%, 3–3.49 mm = 24%, >3 mm = 5%.

**Figure 1:**
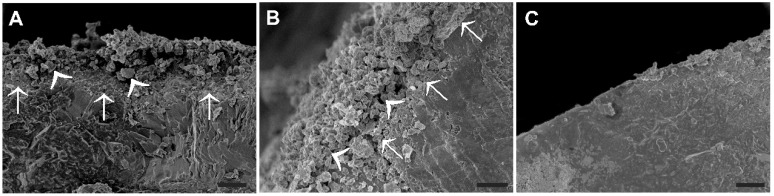
Scanning electron micrographs showing cross sections of fertilizer granules with (**A**,**B**) and without (**C**) an outer layer of sulphur crystals. (**A**) Cross-section of ES coated fertilizer granules containing sulphur crystals of different size (arrowheads) in the outer layer, attached to granule with a binder layer (arrows). (**B**) A bending view of the ES coated fertilizer bead, providing in addition an extended view of its surface covered by ES powder. (**C**) Control granules are characterized by a sharply defined edge without an outer layer. Bars = 20 µm.

Under the SEM, granules of the ES–containing fertilizer were found to be covered with a layer of loose material containing fine crystal-like particles, obviously representing the ES powder layer attached via the binder (Figure 1A,B). The width of the layer was found to be up to 60 µm. Such a layer could not be found on granules of the conventional fertilizer (Figure 1C).

### 3.2. The Effect of the Fertilization Schemes on Yield

Five fields of Group A1 contained no calcium carbonate or traces of it, with pH ranging from 6.20 to 7.74. Four of them characterized by adequate fertility levels, and the FBES-trials presented a relative yield increase of 8.1%, 18.5%, 26.9%, 28.5%, compared to F-trials. In the field FT_07 of Group A1 that was characterized by very low soil initial phosphorus content coupled with low potassium content, the yield of the FBES-trial presented a relative yield decrease (−10.9%) (Table 1 and Table 2).

Five fields of Group A2 contained moderate or high calcium carbonate, with pH ranging from 7.96 to 8.20. In three of them, those with adequate fertility levels, the FBES-trials relative yield increased by 2.7%, 4.2%, 7.8%, respectively. The other two cases of this category presented relative decreases in the yield (−3.4%, −26.8%). The rhizosoil of these cases was characterized by very low initial phosphorus content coupled with marginal iron content, while the rhizosoil of the latter one (−26.8%) additionally presented low concentration of humic substances along with very low sand content (Table 1 and Table 2).

Most of the five fields of Group A3 contained significant amounts of calcium carbonate, with pH ranging from 7.74 to 7.88. In two of them, characterized by adequate fertility levels, the yields of the FBES-trials presented relative yield increases (3.2%, 9.2%). In the fields that characterized by very low soil initial phosphorus content (FT_4, FT_3, FT_5), the yield of the FBES-trial presented a relative yield decrease (−5.9%, −13.0%, −18.0%) (Table 2).

### 3.3. P-Olsen as a Yield-Limiting Factor

**Figure 2:**
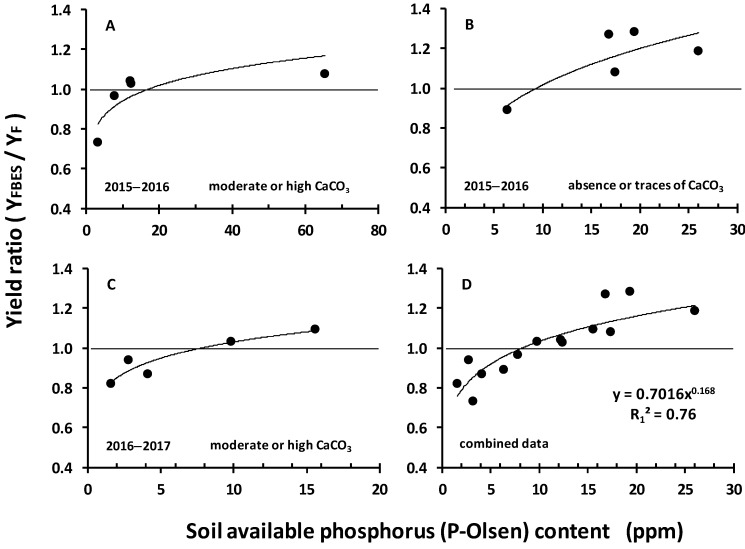
The relationship between the yield ratio (YFBES/YF) and available phosphorus content in the soil. YF, yield of the conventional crop; YFBES, yield of the elemental sulfur treated crop. (**A**) Wheat growing season in 2015–2016: Field trials with moderate of high calcium carbonate content (Group A2). (**B**) Wheat growing season in 2015–2016: Field trials lacking calcium carbonate content (Group A1). (**C**) Wheat growing season in 2016–2017: Field trials with moderate of high calcium carbonate content (Group A3). (**D**) The combination of data.

The P-Olsen content of each field was correlated with the corresponding relative change in the yields (Y_F_/Y_FBES_) and a strong relationship was revealed. This held true for both wheat growing seasons (Figure 2A–C). The season 2015–2016 provided good yields, while the season 2016–2017 provided poor ones. Despite this fact, the relative change due to the application of the elemental sulfur seemed not to be influenced by the Y_F_ level. As a next step the data of both years were combined and fitted by a power function (R_1_^2^ = 0.76, Figure 2D), suggesting that the relationship might not be influenced by the wheat growing season. The level of 8 ppm of available phosphorus was a turning point. At higher values the relative change was higher, i.e., the incorporation of elemental sulfur in the fertilization scheme of the FBES-trial resulted in higher yield compared with the corresponding conventional F-trial, while at lower values the relative change was lower. These results suggested an important role for the soil available phosphorous as a yield-limiting factor.

### 3.4. The effect of ES and NBPT Containing Fertilizers on Yield

**Figure 3:**
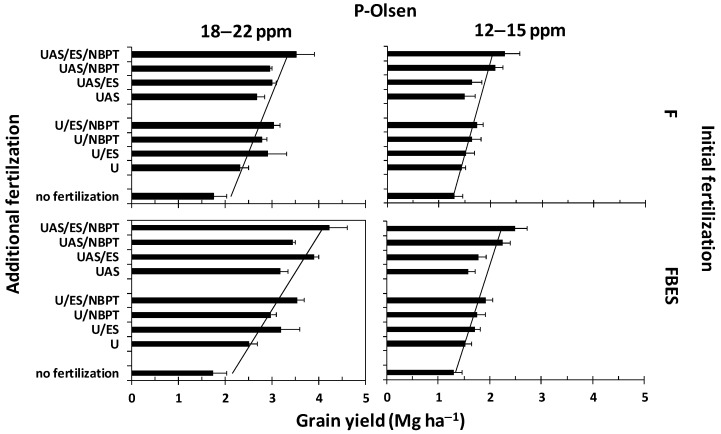
Visualization of the influence of the experiments’ covariates (Phosphorus level, Initial Fertilization and Additional Fertilization) on yield.

The results of experimental field trials supported the aforementioned finding. The corresponding treatments within Group B1 with P-Olsen values ranging between 18–22 ppm (Table 3 and Figure 3) provided higher yields compared with those of Group B2 with P-Olsen values ranging between 12–15 ppm, i.e., less phosphorus by −47% to −50%; (Table 3 and Figure 3). Within each group, the same rank was obtained, i.e., U < UAS < U/ES < UAS/ES. The use of incorporated ES along with the initial fertilization provided statistically higher yields in most cases. This held true within both groups B1 and B2.

The applied fertilizer mixtures that contained both ES and NBPT were more effective in both Groups B1 and B2 compared with their components applied alone (Table 3 and Figure 3). Two types of mixtures were studied: U/ES/NBPT and UAS/ES/NBPT. In Group B1, i.e., when P-Olsen was adequate (18–22 ppm), the rank was U < UAS < U/NBPT < U/ES < UAS/NBPT < UAS/ES < U/ES/NBPT < UAS/ES/NBPT. In any combination of this group, UAS provided higher yields compared to U alone. Furthermore, the incorporation of ES provided higher yields compared to NBPT. The incorporation of elemental sulfur in the initial fertilization provided even higher yields.

In Group B2, i.e., when P-Olsen was marginal (12–15 ppm), the situation altered. In this case the rank was U < UAS < U/ES < UAS/ES < U/NBPT < U/ES/NBPT < UAS/NBPT < UAS/ES/NBPT. Again, UAS provided higher yields compared to U alone. Incorporation of NBPT provided higher yields compared with those of incorporated ES, while UAS/ES/NBPT provided the highest possible yields under the circumstances. Again, the incorporation of elemental sulfur in the initial fertilization provided even higher yields.

In all cases the incorporation of ES in the fertilizer granule provided higher yields than the fertilizer granule alone. Moreover, the 50:50 combination of UAS/ES and UAS/NBPT (UAS/ES/NBPT) provided the highest yields. It is noteworthy that in such a combination actually 1% ES and half the rate of the urease inhibitor were applied to the plot with the additional fertilization.

ANOVA resulted in a model which shows that the linear effects of Phosphorus level (P-Olsen), Initial fertilization (IF) and Additional Fertilization (AF), as well as the pairwise interaction of Phosphorus level with Initial fertilization have a statistically significant effect on the yields’ levels (Table 4 and Table 5).

According to Model results, the overall model predicting value has a total explanatory power (conditional R_2_^2^) of 83.55%, in which the fixed effects explain 82.41% of the variance (marginal R_2_^2^). The model’s intercept was at 2.38 (SE = 0.13, 95% CI [2.14, 2.61]). The effects of low P-Olsen values and especially with no fertilization, UAS/NBPT, and UAS/ES/NBPT were significant and can be considered as large. The effects of UAS/ES, U/ES/NBPT, along with FBES as initial fertilizer were significant and can be considered as medium, while the effects of UAS, U/ES, U/NBPT, as well as the combination low *P*-values along with FBES as initial fertilizer were significant and can be considered as small. The F; U trial served as the reference.

### 3.5. Yield and Head Characteristics

**Figure 4:**
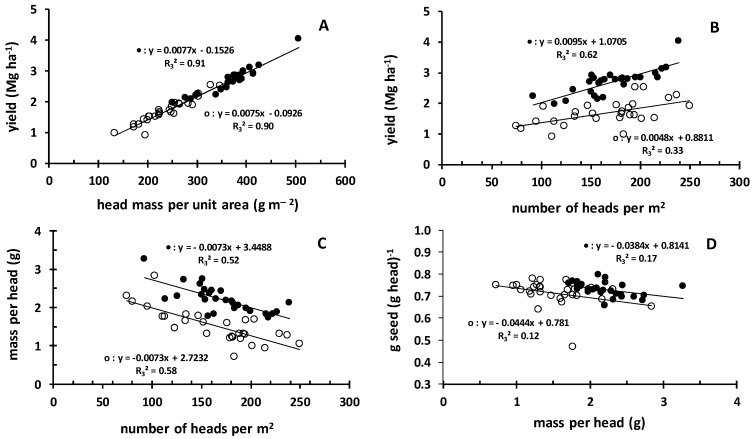
The relationships of the data provided by the treatments of the randomized plot experiments of the 2016–2017 growing season (Group B). Full circles: Data of Group B1 (P-Olsen: 18–22 ppm). Empty circles: Data of Group B2 (P-Olsen: 12–15 ppm). In each set of data, the tendency is described by including a trend line of linear type.

Yield presented a positive linear relationship with head mass per square meter (Figure 4A), with the same slope for both Groups B1 and B2. This held true for the correlation of yield with the number of heads per square meter (Figure 4B), however in this correlation Group B1 was separated from Group B2. The mass per head presented a negative linear relationship with the number of heads per square meter (Figure 4C), and again Group B1 was separated from Group B2. Such a negative linear relationship was also revealed when mass per head was correlated with the ratio of seed mass per head mass (Figure 4D). Again, the two groups were separated, however the slope was not steep.

## 4. Discussion

### 4.1. The Role of Soil Available P on the Relative Yield

The results of the relative yields were contradictory, as some FBES-trials provided lower yields compared to the corresponding conventional ones. It was further shown that soil available phosphorus was a strong limiting factor and the P-Olsen value of 8 ppm was established as the turning point. Above this threshold the relative yield was increased.

Sulfate-S is the available S source to the plants, while ES as S source is not readily available to plants. The conversion of ES to sulfate requires the action of microorganisms [27]. Recent studies showed that S-oxidizing bacteria activity depends on the variations in soil organic C and nutrient availability [28]. In a recent study, it was concluded that in soils with pH > 6.65, along with high S and organic matter content, the ES oxidation rate is higher compared to the other soils [29]. According to our results, it is speculated that the low organic matter content and the low available phosphorus are limiting factors for the activity of S-oxidizing rhizosoil microbes, the growth of which is boosted by the added ES with the fertilizer granule. We have shown that the application of FBES fertilizers increases the total numbers of microbial populations in the rhizosphere [30]. In the initial experiment [13], the 20-10-10/ES that was applied at sowing contained both ES and sulfate, i.e., both readily available and non-available S to plants, thus supporting crop growth with sulfate for an extended period. The role of soil microbes in the biogeochemical S cycle and S supply of plants has been reviewed; fungi and bacteria release S from sulfate-esters using sulfatases [31,32], while the interactions of roots with soil microorganisms, especially non-symbiotic plant growth promoting rhizobacteria, in relation to nutrient availability, as well as the mechanisms that are associated with plant growth promotion have been discussed [33]. We have found that a portion of the existing microorganisms in the rhizosphere presented arylsulfatase activity [30], thus releasing sulfate bound in the soil organic matter, the percentage of which increased with the addition of ES-enriched fertilizer. Moreover, the fertilization with FBES at sowing affected the iron fractions of the rhizosoil towards iron mobilization, thus providing more iron to the crop, which apart from the iron nutrition fortified the crop’s sulfur nutrition, too [14].

On the other hand, phosphorus (P) is one of the most limiting essential nutrients for crop production. The dynamic processes determining P availability in the soil and in the rhizosphere, P mobilization, uptake, and utilization by plants have been reviewed [34,35]. Especially, the response of wheat grain yield to phosphorus has been reported by a number of researchers, as wheat crops have high phosphorus requirement, mostly during the early growth stages [36,37,38,39]. Although yield limiting factors are complex, the effect of soil available P was studied further in experimental plots under a randomized block design. Already existing spatial information of the experimental site [40] was used to guide sampling to representative plots, referred to in the literature as directed, smart or targeted sampling [41]. In accordance with Friesen (1996) [42], our results suggested that the presence of adequate P for the needs of the S-oxidizing bacteria may be a critical factor for the ES use efficiency. It is speculated that under low soil available P, the increased numbers of microbial populations, due to the addition of ES in the fertilization scheme were stronger P consumers compared to the wheat crop during the growing season, leaving less than adequate P for the crop’s needs, thus possibly explaining the depressed yields. More research is needed to fully explain the effect of soil available P on yield under the circumstances.

### 4.2. The Efficacy of ES Fertilization Schemes

ES is used either as granules, or in the form of fine powder, the micronized ES. Micronized ES is preferable compared with ES granules, because the transformation to sulfate-S by the microorganisms is quick. On the other hand, the application of fine powder in large scale agriculture is a serious problem, because the handling of dust is involved. To overcome the problem, several technologies towards successful co-formulation of micronized ES with different materials have been arisen through the years, in order to produce granules applicable to large scale agriculture, including monoammonium phosphate, diammonium phosphate, and triple superphosphate, containing micronized ES without or with AS [43,44]. Scanning electron migrographs of 20-10-10/ES (2%) granules clearly show that elemental sulfur forms a dense coating layer on the surface, which is achieved via the sticky mixture of molasses plus glycerol. The product 20-10-10/ES produced dust. This held true for 16-20-0/ES(2%), too. The dust was estimated to be in the order of 4%, a minor fraction of which was ES (data not shown). On the other hand, U/ES(2%) and UAS/ES(2%) were indeed dustless.

The factors that influence the oxidation of elemental sulfur in soils have been discussed [27], while a “concept of the negative locality effect on ES oxidation” has been developed [8,44]. Briefly, when the fertilizer granule disintegrates it releases micronized ES particles to the soil. Due to the hydrophobic nature of the ES, the very fine ES particles create clusters and localize around the applied granule site. Clustering decreases contact between localized ES particles and soil, which in turn decreases the colonization of soil S-oxidizing bacteria on the surface of the ES particles. Moreover, the released micronized ES particles can coalesce to form larger aggregates, which in turn can further decrease ES oxidation [42,45]. It seems that the FBES technology diminishes the negative locality effect on ES oxidation. The binder utilized by the FBES technology is also a positive factor, because the binder around the ES and the granule particles can be consumed by the microorganisms as well.

The fertilizer type 16-20-0 was used and tested against 16-20-0/ES as NP starter fertilizer, which was combined with U or UAS, applied in spring as topdressing added N (and S). U and UAS are of common use and both water soluble. N from urea is available to the plant only after hydrolysis by the urease enzyme to ammonium carbonate, which can significantly increase soil pH around the applied urea granule sites (can be as high as 8–9), due to hydroxyl anion production. AS provides N and S, both critical plant nutrients. Compared with urea, AS may have some potential agronomic and environmental benefits [44]. One approach to enhance the N efficiency of urea is to partially substitute AS for urea in the mixture. The dilution effect of AS-N should be considered because ammonia volatilization is greater with increased urea concentration and/or rate of application [46]. An increase in crop yield should be expected by mixing AS and urea compared with urea alone. Another reason to mix AS and urea is to supply S along with N. Moreover, AS reacts with calcium carbonate to precipitate calcium sulfate. Sulfur-coated urea (SCU) as a source of S was used in wheat crop and it was found that 5% of SCU supplied 50% of the S wheat requirements and at the same time increased N recovery efficiency by 60.3% over prilled urea [47]. Our results showed that the incorporation of ES improved the performance of U or UAS, suggesting that the granules enriched with ES were fortified, while their performance was influenced by the soil available phosphorus.

### 4.3. The Efficacy of ES and NBPT Fertilization Schemes

The stability of urea in the soil is affected by the enzyme urease, which is released by the soil microbial population or is derived from the decomposition of organic matter [11]. The incorporation of urease inhibitors into urea granules delays urea hydrolysis and urea is available for plant acquisition. N-(n-butyl) thiophosphoric triamide (NBPT) is the urease inhibitor that is often formulated into granular urea fertilizers. This compound is a structural analog of urea and inhibits urease activity by forming stable complexes with urease [11,48,49]. The use of granules co-enriched with ES and NBPT onto the same granule in a field experiment has been reported [50]. According to the authors, the inhibitor and ES were coated onto the fertilizers by Summit-Quinphos Ltd. (New Zealand) and applied directly onto the pasture of the respective plots by hand. No information was provided on the nature of the coating. In the present study, we tested the efficacy of a 50:50 mixture of NBPT containing granules and ES containing granules, using commercially available fertilizers produced by the same company. The 50:50 ratio was arbitrarily chosen. Such combination provided even higher yields compared to the yields provided by the application of the corresponding fertilizers alone and the soil available phosphorus influenced the performance of the mixture. According to the FBES technology, the core fertilizer can be enriched up to 4% with ES. To our knowledge, the least percentage of ES in NP fertilizer granules reported in the literature is 5% [10]. In this research, granules enriched with 2% ES were used, i.e., the minimum ES enrichment. Therefore, in the 50:50 mixture, more or less 1% of ES was applied, i.e. half of the amount, and the same holds true for NBPT.

In summary, UAS provided sulfate-S for immediate use, ES as a coating in its majority provided sulfate-S after oxidation, NBPT positively contributed to ammonium use efficiency provided by both U and AS constituents of the fertilizer granule, while the minor amount of the binding materials probably fed the microorganisms around the granule. A portion of these microorganisms were arylsulfatase producing ones, which implies that organic S was released as sulfate-S, thus comprising a third pool of sulfate-S. The more the available P the more effective the UAS/ES/NBPT mixture was. The efficacy of this activity was enhanced when FBES (16-20-0/ES) was used as starter fertilizer at sowing, instead of the corresponding 16-20-0 conventional fertilizer.

## 5. Conclusions

The use of UAS in spring applied fertilization scheme enriched with 2% ES and the urease inhibitor NBPT in the granules, significantly increased the yield of durum wheat crops, and in combination with the use of 2% ES as an ingredient of the starter fertilizer, it boosted the yield to even higher scores. The soil available phosphorus at P-Olsen value of 8 ppm seemed to be the threshold, below which the performance of the aforementioned combination was strongly depressed.

## Figures and Tables

**Table 1:** Fertilization schemes per trial. FT, field trial; C, field trial with the conventional fertilization (F-)treatment, S, the corresponding field trial with incorporated elemental sulfur in the fertilization scheme (FBES-treatment); F1, initial fertilization along with sowing; F2a, first additional fertilization; F2b, second additional fertilization; Δx/x, percentage relative change of grain yield; 46-0-0, urea; 40-0-0, urea plus ammonium sulfate; 34.5-0-0, ammonium nitrate; /ES, fertilizer granules with incorporated 2% elemental sulfur.

Group	Field Trial	Area ha	Seed kg ha^−1^	Cultivar	F1 kg ha^−1^	Fertilizer Type	F2a kg ha^−1^	Fertilizer Type	F2b kg ha^−1^	Fertilizer Type	Δx/x (%)
	**2015–2016**										
**A1**	FT_13C	1.4	280	Meridiano	250	16-20-0	120	46-0-0			
	FT_13S	1.4	280	Meridiano	250	16-20-0/ES	120	46-0-0			28.5
	FT_08C	1.4	250	Quadrato	300	20-10-0	200	40-0-0			
	FT_08S	1.4	250	Quadrato	300	20-10-0/ES	200	40-0-0			26.9
	FT_03C	1.5	250	Quadrato	300	16-20-0	250	40-0-0			
	FT_03S	1.5	250	Quadrato	300	16-20-0/ES	250	40-0-0/ES			18.5
	FT_12C	1.35	250	Simeto	250	10-20-0	100	46-0-0	100	34.5-0-0	
	FT_12S	1.35	250	Simeto	250	10-20-0/ES	100	46-0-0	100	34.5-0-0	8.1
	FT_07C	1.4	220	Normano	280	20-10-0	250	45-0-0			
	FT_07S	1.4	220	Normano	280	20-10-0/ES	250	45-0-0			−10.9
**A2**	FT_02C	1.0	250	Quadrato	300	16-20-0	100	40-0-0	150	34.5-0-0	
	FT_02S	1.0	250	Quadrato	300	16-20-0/ES	100	40-0-0	150	34.5-0-0	7.8
	FT_10C	1.1	280	Meridiano	300	16-20-0	220	40-0-0			
	FT_10S	1.1	280	Meridiano	300	16-20-0/ES	220	40-0-0			4.2
	FT_11C	1.5	280	Normano	300	16-20-0	300	40-0-0			
	FT_11S	1.5	280	Normano	300	16-20-0/ES	300	40-0-0			2.7
	FT_01C	0.9	250	Simeto	275	16-20-0	180	40-0-0	180	40-0-0	
	FT_01S	0.9	250	Simeto	275	16-20-0/ES	180	40-0-0/ES	180	40-0-0/ES	−3.4
	FT_09	1.5	250	Meridiano	300	16-20-0	200	40-0-0			
	FT_09	1.5	250	Meridiano	300	16-20-0/ES	200	40-0-0/ES			−26.8
	**2016–2017**										
**A3**	FT_6C	1.3	280	Meridiano	300	16-20-0	200	40-0-0			
	FT_6S	1.3	280	Meridiano	300	16-20-0/ES	200	40-0-0/ES			9.5
	FT_7C	1.5	280	Meridiano	300	16-20-0	200	40-0-0			
	FT_7S	1.5	280	Meridiano	300	16-20-0/ES	200	40-0-0/ES			3.2
	FT_3C	1.5	280	Meridiano	300	16-20-0	200	40-0-0			
	FT_3S	1.5	280	Meridiano	300	16-20-0/ES	200	40-0-0/ES			−13.0
	FT_4C	0.7	250	Simeto	300	20-10-0	230	45-0-0			
	FT_4S	0.7	250	Simeto	300	20-10-0/ES	230	45-0-0			−5.9
	FT_5C	0.9	250	Simeto	300	20-10-0	220	40-0-0			
	FT_5S	0.9	250	Simeto	300	20-10-0/ES	220	40-0-0			−18.0

**Table 2:** Soil analysis data and yields. OM, soil organic matter; HS, soil humic substances; YF, yield of the conventional crop; YFBES, yield of the elemental sulfur treated crop; Δx/x, YFBES percentage change relative to YF.

Group	Field Trial	Sand (%)	Silt (%)	Clay (%)	Type	pH	ECe μS/cm	NO_3_^−^N mg/kg	YF	YFBS	Δx/x (%)
									Mg ha^−1^		
	**2015–2016**										
**A1**	FT_13	34	30	36	CL	6.20	479	20.2	5.19	6.67	28.5
	FT_08	24	30	46	C	7.53	505	16.0	3.60	4.57	26.9
	FT_03	26	24	50	C	6.95	352	11.5	4.65	5.51	18.5
	FT_12	22	44	34	CL	7.17	603	25.7	4.30	4.65	8.1
	FT_07	22	44	34	CL	6.65	386	17.1	4.60	4.10	−10.9
**A2**	FT_02	28	40	32	CL	8.02	572	15.6	6.16	6.64	7.8
	FT_10	28	34	38	CL	8.10	453	9.1	4.30	4.48	4.2
	FT_11	34	38	28	CL	7.96	675	16.6	4.40	4.52	2.7
	FT_01	28	30	42	C	8.01	613	24.6	5.06	4.89	−3.4
	FT_09	10	42	48	SiC	8.00	592	23.2	3.32	2.43	−26.8
	**2016–2017**										
**A3**	FT_6	30	34	36	CL	7.81	667	23.1	1.68	1.84	9.5
	FT_7	14	40	46	SiC/C	7.88	610	25.5	4.73	4.88	3.2
	FT_3	16	38	46	C	7.74	491	10.5	2.62	2.28	−13.0
	FT_4	26	28	46	C	7.80	684	19.6	1.53	1.44	−5.9
	FT_5	30	28	42	C	7.74	571	19.9	1.78	1.46	−18.0
**B1**		23	25	52	C	8.57	543	19.2			
**B2**		23	24	53	C	8.13	527	17.1			
	**Field**	**CaCO_3_**	**P-Olsen**	**Kexch**	**Fe-DTPA**	**Mn-DTPA**	**Cu-DTPA**	**Zn-DTPA**	**SOM**	**HS**	**HS/SOM**
	**Trial**	**(%)**	**mg/kg**	**mg/kg**	**mg/kg**	**mg/kg**	**mg/kg**	**mg/kg**	**(%)**	**(%)**	**(%)**
	**2015–2016**										
**A1**	FT_13	0	19.4	230	22.7	13.4	1.5	0.8	1.5	0.2	11.7
	FT_08	0	16.8	300	17.7	12.1	1.5	0.7	2.3	0.5	21.1
	FT_03	0	26.0	230	26.3	13.4	4.7	1.8	1.8	0.2	12.7
	FT_12	0	17.4	270	25.8	12.2	2.2	1.8	1.9	0.2	8.1
	FT_07	0	6.4	180	27.3	13.4	2.2	0.7	2.3	0.3	14.3
**A2**	FT_02	5.2	65.4	240	13.0	8.4	1.5	1.1	2.1	0.2	9.9
	FT_10	49.1	12.2	390	15.9	9.3	1.8	0.7	1.8	0.1	2.8
	FT_11	6.6	12.4	270	11.2	7.3	1.1	0.7	1.8	0.1	5.0
	FT_01	24.5	7.8	270	5.8	6.3	1.4	0.8	1.8	0.1	6.7
	FT_09	26.6	3.2	290	8.0	6.2	1.3	0.5	2.9	0.1	2.4
	**2016–2017**										
**A3**	FT_6	41.0	15.6	450	5.8	9.6	1.7	0.8	2.4	0.1	4.2
	FT_7	1.4	9.8	350	14.8	10.1	1.5	0.7	3.7	0.4	10.8
	FT_3	13.9	4.1	420	12.6	7.5	1.2	0.5	3.0	0.2	7.8
	FT_4	11.1	2.8	240	10.1	7.3	0.9	0.9	1.9	0.1	7.2
	FT_5	8.9	1.6	240	8.0	6.4	0.7	0.9	2.1	0.2	9.4
**B1**		18.1	20.2	230	24.3	12.1	1.8	0.9	2.5	0.2	9.6
**B2**		21.7	13.1	224	21.3	11.7	2.0	0.9	2.3	0.2	9.9

**Table 3:** Results of the randomized plot experiments of 2016–2017 growing season (Group B). F (16-20-0) or FBES (16+20-0+2%ES): Initial fertilization (F1) along with sowing, U, urea 46-0-0; UAS, urea plus ammonium sulfate 40-0-0. “/NBPT” denotes fertilizer with NBPT (N-(n-butyl) thiophosphoric triamide; urease inhibitor). “/ES” denotes fertilizer with 2% elemental sulfur. “/NBPT/ES” denotes application of a 50:50 mixture of fertilizer with NBPT and the same fertilizer with 2% elemental sulfur. The F; U trial served as a reference, with rates of F1 and F2 (additional fertilizer application) 250 kg ha^−1^ respectively. Simeto cultivar was used.

**Treatment**	**Group B1**			**Group B2**			**Group B1**		**Group B2**	
	P-Olsen (ppm)						P-Olsen (ppm)			
	18–22			12–15			18–22		12–15	
	Grain yield (Mg ha^−1^)						Yield relative to the combination F; U (%)			
no fertilization	1.75			1.30			−25		−10	
	F	FBES	RC (%)	F	FBES	RC (%)	F	FBES	F	FBS^0^
U	2.32	2.51	8.2	1.44	1.52	5.6	0	8	0	6
U/ES	2.90	3.19	10.0	1.53	1.71	11.8	25	38	6	19
U/NBPT	2.78	2.98	7.2	1.64	1.76	7.3	20	28	14	22
U/NBPT/ES	3.04	3.54	16.4	1.74	1.93	10.9	31	53	21	34
UAS	2.68	3.18	18.7	1.50	1.58	5.3	16	37	4	10
UAS/ES	3.00	3.90	30.0	1.64	1.78	8.5	29	68	14	24
UAS/NBPT	2.95	3.45	16.9	2.09	2.25	7.7	27	49	45	56
UAS/NBPT/ES	3.52	4.22	19.9	2.27	2.49	9.7	52	82	58	73

**Table 4:** Analysis of Variance with Satterthwaite’s method. P-Olsen: IF denotes the interaction between P-Olsen and IF.

Variable	Sum Sq	Mean Sq	NumDF	DenDF	*P*-Value
P-Olsen	11.6159	11.6159	1	87	<0.001
IF	3.0111	1.5056	2	87	<0.001
AF	10.5053	1.5008	7	87	<0.001
P-Olsen:IF	1.7335	0.8667	2	87	0.0013

**Table 5:** Regression coefficients (95% confidence intervals) and *p*-values for the selected model. (IF, initial fertilization; AF, additional fertilization; NF, no fertilization)

Variable	Beta	95% CI		*P*-Value
Intercept	2.38	2.14	2.61	<0.001
P-Olsen; Low	−1.17	−1.35	−0.98	<0.001
AF; UAS/ES/NBPT	1.18	0.91	1.44	<0.001
AF; UAS/NBPT	0.74	0.47	1.00	<0.001
P-Olsen; Low: IF; NF	0.72	0.16	1.28	0.019
AF; UAS/ES	0.63	0.37	0.90	<0.001
IF; FBES	0.47	0.29	0.66	<0.001
IF; NF	−0.63	−1.06	−0.19	0.009
AF; U/ES/NBPT	0.61	0.35	0.88	<0.001
AF; U/ES	0.38	0.12	0.65	0.008
AF; U/NBPT	0.34	0.08	0.61	0.018
AF; UAS	0.29	0.02	0.55	0.046
P-Olsen; Low: IF; FBES	−0.33	−0.59	−0.06	0.024

## Abbreviations

RS	rhizosoil
OM	organic matter
HS	humic substances
F	granular conventional fertilizer
ES	elemental sulfur
B	binder
U	urea
UAS	urea ammonium sulfate
NBPT	N-(n-butyl) thiophosphoric triamide
